# Characterization of a highly divergent *Sugarcane mosaic virus* from *Canna indica* L. by deep sequencing

**DOI:** 10.1186/s12866-019-1636-y

**Published:** 2019-11-21

**Authors:** Yongqiang Li, Fei Xia, Yixuan Wang, Chenge Yan, Anning Jia, Yongjiang Zhang

**Affiliations:** 1 Beijing Key Laboratory of New Technology in Agricultural Application, National Demonstration Center for Experimental Plant Production Education, Beijing University of Agriculture, Beijing, China; 2 Key Laboratory for Northern Urban Agriculture of Ministry of Agriculture and Rural Affairs, Beijing University of Agriculture, Beijing, China; 3 Beijing Institute of Landscape Architecture, Beijing, China; 4 Chinese Academy of Inspection and Quarantine, Beijing, China

**Keywords:** Deep sequencing, Sugarcane mosaic virus, Canna

## Abstract

**Background:**

Cannas are popular ornamental plants and widely planted for the beautiful foliage and flower. Viral disease is a major threaten to canna horticulture industry. In the city of Beijing, mosaic disease in canna was frequently observed, but the associated causal agent and its biological characterization is still unknown.

**Results:**

After small RNA deep sequencing, 36,776 contigs were assembled and 16 of them shared high sequence identities with the different proteins of *Sugarcane mosaic virus* (SCMV) of the size ranging from 86 to 1911 nt. The complete genome of SCMV isolate (canna) was reconstructed by sequencing all cDNA clones obtained from RT-PCR and 5′\3′ RACE amplifications. SCMV-canna isolate showed to have a full RNA genome of 9579 nt in length and to share 78% nt and 85% aa sequence identities with SCMV isolates from other hosts. The phylogenetic tree constructed based on the full genome sequence of SCMV isolates allocated separately the canna-isolate in a distinct clade, indicating a new strain. Recombination analyses demonstrated that SCMV-canna isolate was a recombinant originating from a sugarcane-infecting isolate (major parent, acc. no. AJ310103) and a maize-infecting isolate (minor parent, acc. no. AJ297628). Pathogenicity test showed SCMV-canna could cause typical symptoms of mosaic and necrosis in some tested plants with varying levels of severity but was less virulent than the isolate SCMV-BJ. Field survey showed that the virus was widely distributed.

**Conclusions:**

This study identified SCMV as the major agent causing the prevalent mosaic symptom in canna plants in Beijing and its genomic and biological characterizations were further explored. All these data enriched the knowledge of the viruses infecting canna and would be helpful in effective disease management in canna.

## Background

*Canna indica*, a member in the family *Cannaceae*, was listed among the most popular ornamental plants [1, 2] for the beautiful foliage and flower grown in tropical and temperate regions [3, 4]. As other flowering plants, the economic value of canna plants was often affected especially by viral disease due to the vegetative propagation (rhizome cuttings) and trade of untested material. Five viruses have been isolated from canna: two potyviruses-*Bean yellow mosaic virus* (BYMV) and *Canna yellow streak virus* (CaYSV), two cucumoviruses-*Cucumber mosaic virus* (CMV) and *Tomato aspermy virus* (TAV), and a DNA virus-*Canna yellow mottle virus* (CaYMV, family *Caulimoviridae*, genus *Badnavirus*), among which CaYSV and CaYMV were the most widely described.

CaYSV was a newly reported potyvirus first identified in 2007 responsible for the symptoms of speckling and streaking on the leaves and had been found in UK, Belgium, Netherlands, France, Israel, USA and Russia [5, 6]. Sequence analyses showed that CaYSV belongs to the *Sugarcane mosaic virus* subgroup with *Johnsongrass mosaic virus* as its closest related-virus [7, 8]. CaYMV, often present in mixed infection with CMV, was first reported in the 1980s in Japan and North America and now has expanded to Italy, the Netherlands, India, Kenya, Russia and other regions of the United States [6, 9–15]. Recent studied indicated CaYMV has expanded its host range to infect *Alpinia* in Hawaii and *Piper betel* L*.* in India [16, 17]. Once infected, canna plants often appear leaf colouring [7, 8, 18]. These viruses were typically reported as single infections, while CaYMV and CaYSV often appeared in the same plant.

RNA silencing is a conserved mechanism for plants to defend against virus infection. Once infected by viruses in plants, the RNA silencing elements associated with AGO, DCL and RdRp would cleave the virus genome into small RNAs with the length ranged from 18 to 28 nts termed as virus-derived small interfering RNAs (vsiRNAs). The whole genome was the potential cleaved targets, thus the vsiRNAs were overlapped in sequences and could be assembled into contigs even complete virus genome, which supplied an alternative method for plant virus identification. With this method, many new or known plant viruses with different types of genomes were characterized [19–23].

In this study, small RNA deep sequencing analysis of canna leaves with mosaic symptom was performed to identify the causal agent associated with this disease. Sequence assembly and blast analyses showed a potyvirus present in the collected sample. The complete genome of this virus was gained with conventional RT-PCR and Rapid Amplification of cDNA Ends (RACE) PCR for both ends. The virus was identified as a *Sugarcane mosaic virus* isolate, highly divergent from other isolates.

## Results

### sRNA sequence analyses and contigs assembly

A total of 21,800,471 reads was sequenced in the sample and after quality trimming 21,394,724 clean reads with the dominant size of 21-, 22- and 24 nt were obtained. The corresponding clean reads were de novo assembled, resulting in 36,776 contigs with the size ranging from 18 to 1911 nt and annotated by BLASTn and BLASTx analysis against the GenBank database. Most of the contigs were host-originated while 16 contigs ranging from 86 to 1911 nt with high levels of identity with the different proteins of SCMV by BLASTx were exclusively identified. The overlapped contigs were further manually assembled by mapping against a reference SCMV genome (AY042184). Finally the full genome sequence of SCMV-canna were finally completed with RACE PCR for both ends of the virus genome and conventional RT-PCR with overlapped specific primer pairs for the internal gaps (Additional file 3: Table S1) with the GenBank accession number KU561096.The genome sequence was further confirmed in one PCR reaction with the primer pair P1 F and CP R in Additional file 3: Table S1.

### Sequence analysis of SCMV-canna

**Fig. 1:**
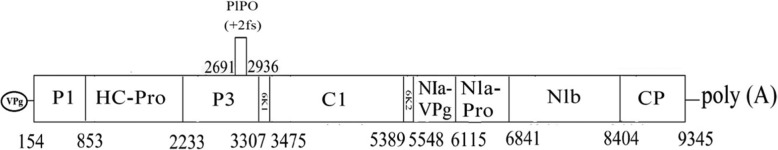
Schematic representation of the genomic organization of SCMV-canna. The 5′ and 3′ nontranslated regions (NTR) are represented by lines, and the large open reading frame (ORF) is depicted by an open box. The numbers below the diagram indicate the starting position (nt) predicted for each gene. The position of PIPO was also listed

**Fig. 2:**
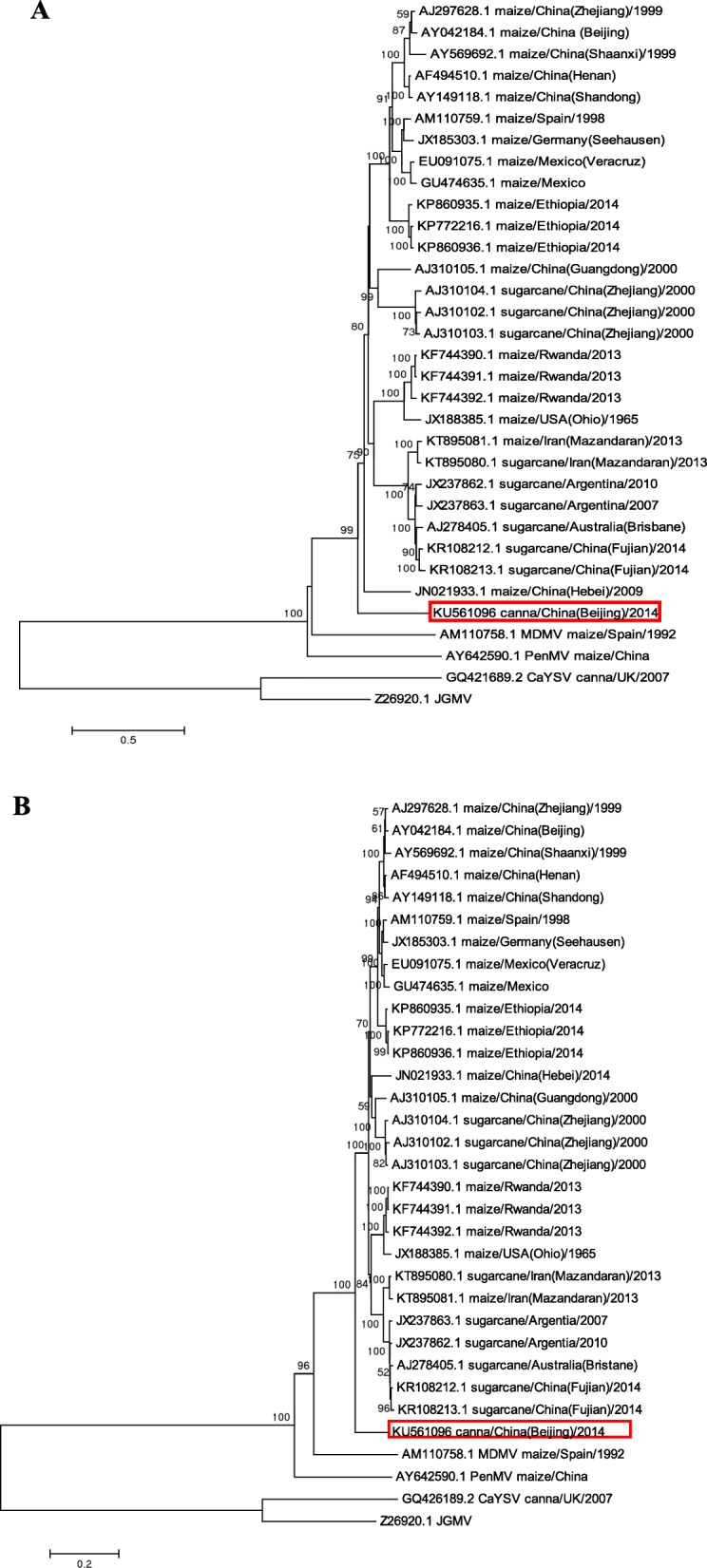
Neighbor-joining trees obtained from alignment of the complete genome nucleotide sequence (**a**) and amino acid sequence (**b**) of SCMV with 1000 bootstrap replicates. Bootstrap values are given by numbers at the relevant nodes in the topology and only bootstrap values of ≥50% were shown. Maize dwarf mosaic virus (MDMV), *Pennisetum mosaic virus* (PenMV), *Canna yellow streak virus* (CaYSV), *Johnsongrass mosaic virus* (JGMV) were used as outgroups

This SCMV-canna isolate was 9579 nt in length, excluding the poly (A) tail at the 3′ end with the 5′ and 3′ untranslated regions (UTR) of 153 and 234 nt, respectively. Like other potyviruses, SCMV-canna contained a large open reading frame (ORF, nt 154–9345) encoding a polyprotein of 3063 amino acid (aa) residues of 346.8 kDa. BLAST analysis showed SCMV-canna shared 78% nt and 85% aa sequence identities with SCMV isolates present in the GenBank. Protein sequence analysis based on the BLASTX results (Fig. 1) identified nine putative cleavage sites for the ten mature protein (P1, HC-Pro, P3, 6 K1, CI, 6 K2, NIa-VPg, NIa-Pro, NIb and CP) at amino acid positions 233, 693, 1040, 1107, 1745, 1798, 1987, 2229 and 2750. The recently identified ORF coding the putative protein PIPO [24] was identified from a G2A6 motif at position 2691within the P3 region protein in the + 2 reading frame. Typical motifs reported in potyviruses were found in the corresponding amino acid sequences of these mature proteins: the serine-type protease domain^153^H-8X-^162^D-31X-^194^G-X-^196^S-^197^G and the proteolytic domain ^207^FIVRGR^212^ in P1; ^579^C-72X-H^652^ in HC-Pro, and ^2033^H-34X-D^2068^-69X-C^2138^ in NIa-Pro [25], the conserved motifs ^287^KITC^290^, ^545^PTK^547^, and ^416^FRNK^419^ in HC-Pro and ^2755^DAG^2757^ in CP that were indispensable for aphids transmission [26, 27], the conserved motifs ^527^CCCVT^531^ in HC-Pro and ^2952^R-X43-D^2996^ in CP involving in the virus long-distance movement; the nucleotide-binding motif ^1195^GAVGSGKST^1203^ and the RNA helicase domains ^1284^DEXH^1287^, ^1215^VLLIEPTRPL^1224^, ^1311^KVSAT^1315^, ^1362^LVYV^1365^, ^1413^VATNIIENGVTL^1424^ and ^1457^GERIQRLGRVGR^1468^ in CI [28, 29], the motif ^1860^MYGF^1863^ in which the tyrosine linked VPg to the 5′-end of the genomic RNA [30], and the RNA-dependent RNA polymerase motifs ^2475^CDADGS^2480^ and^2540^SG-X3-T^2545^-X3-NT^2550^-X30-G-D-D^2583^ in NIb [29]. To elucidate the phylogenetic relationship with other isolates, phylogenetic tree was constructed with the SCMV complete genome nt/aa sequences available in the GenBank database with NJ method respectively. SCMV-canna formed separately in the phylogenetic trees (Fig. 2a and b), indicating its distinct phylogenetic relationship with other SCMV isolates. The topology was further verified by ML method based phylogenetic analyses (Additional file 1: Figure S1A and B). CP sequences were a good index of genetic relatedness and potyviruses classification criteria [31], thus to further characterize the genetic relationship of SCMV-canna, CP-based phylogenetic tree was constructed and SCMV-canna clustered together with the sugarcane isolates from Yunnan of China and Viet Nam (Additional file 2: Figure S2). Recombination analyses showed SCMV-canna was a recombinant identified by the software RDP, MaxChi and Chimaera (with the *P*-value of 1.840 × 10^− 2^, 2.479 × 10^− 2^ and 2.465 × 10^− 3^ respectively and within the region of nt 3878–4343) originating from a sugarcane-infecting isolate with the acc. no. AJ310103 as its major parent and a maize-infecting isolate with the acc. no. AJ297628 as its minor parent, indicating its origin from sugarcane-infecting isolate.

### Field survey for the presence of SCMV

To survey the epidemiology of this virus in canna, 64 canna plants with mosaic symptom originating from different districts of Beijing were assayed using RT-PCR detection with a primer pair (Additional file 3: Table S1) targeting the CP gene. Results of RT-PCR detection showed that SCMV was present in nearly all the samples (63/64), indicating the prevalence of SCMV in canna.

### Infectivity of SCMV-canna in tested plants

**Fig. 3:**
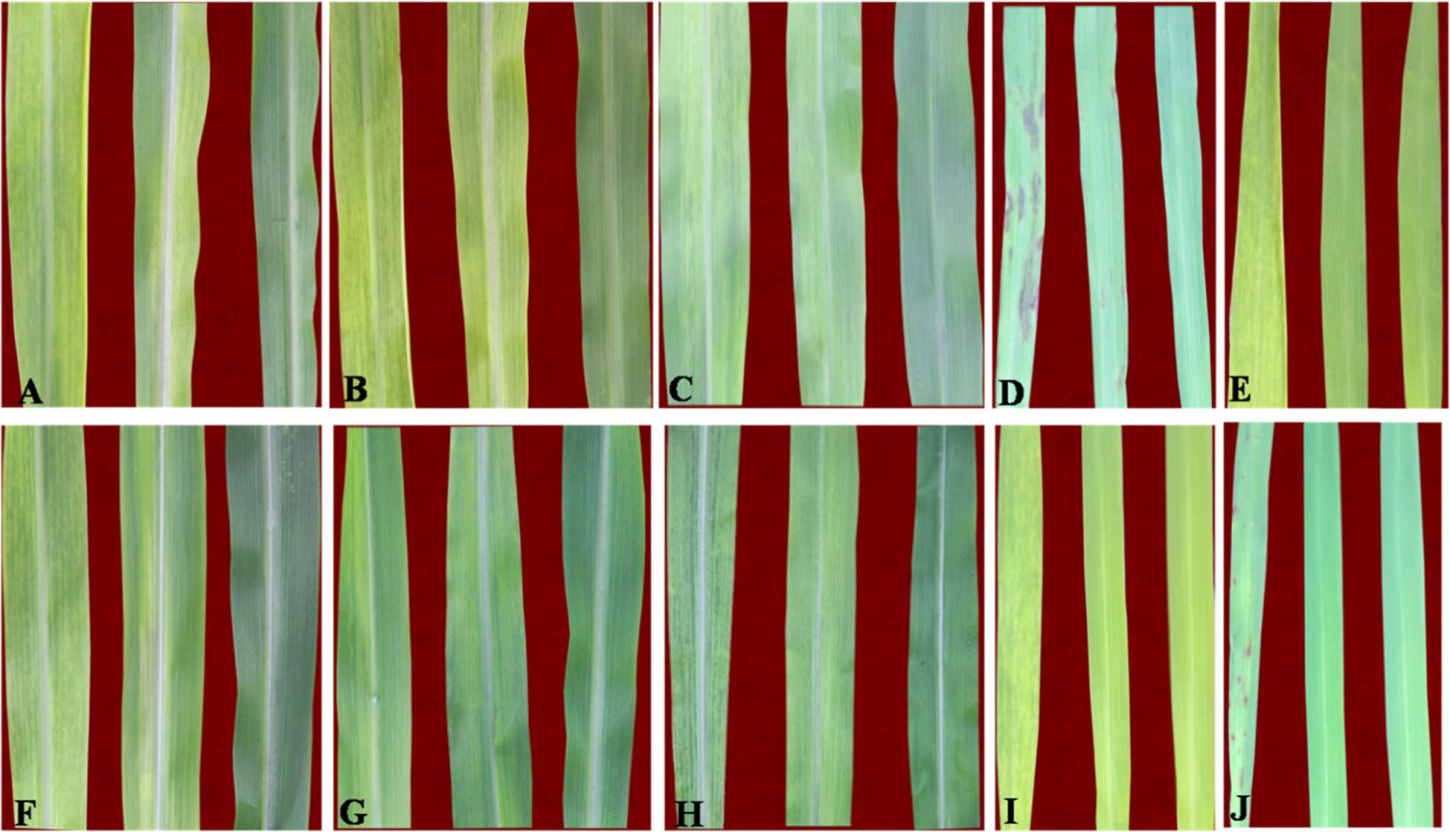
Pathogenicity test of SCMV-canna on different plants. Maize cultivars **a** denghai 605, **b** ludan 6006, **c** ludan 9066, **f** luke super maize, **g** jundan 20, **h** nongda 2238; **d** *Pennisetum alopecuroides* (L.), **e** *Pennisetum purpureum* Schum., **i** Sorghum Hybrid sudan grass, **j** *Sorghum sudanense (Piper) Stapf.*. In each cultivar, the left was infected with SCMV-BJ, while the middle with SCMV-canna and the right with PBS buffer

As other SCMV isolates, SCMV-canna could not infect the plants *Nicotiana benthamiana*, *Nicotiana tabacum* (cv. Xanthi nc), *Chenopodium amaranticolor* and *C. quinoa*. Typical mosaic symptom could be observed on all the tested maize cultivars (Fig. 3a-c, f-h) while necrotic lesions appeared on the *Pennisetum alopecuroides* (L.) (Fig. 3d), which was less severe than that caused by the isolate SCMV-BJ. Interestingly, no symptoms was observed in SCMV-canna inoculated *Sorghum sudanense (Piper) Stapf.*, Sorghum Hybrid sudan grass and *Pennisetum purpureum* Schum. plants while typical visible symptoms were caused by SCMV-BJ. These results were further confirmed with RT-PCR.

## Discussion

The development of next generation sequencing (NGS) technologies has greatly advanced our ability to comprehensively investigate the aetiology of viral disease [23]. In this work, a highly divergent SCMV isolate was identified from canna leaf samples and the full genome sequence was determined using a combination of bioinformatic analysis and RT-PCR based sequencing to fill gaps in the contigs assembled from the sequencing data, which supplied a rapid and precise method for the agents underlying the mosaic disease in canna.

The ability to adapt to new hosts is an important biological property of most RNA viruses, which is particularly important for plant viruses that infect annual crop species with relatively short life and therefore need to constantly infect and adapt to new plant hosts. SCMV is a definitive member of the genus *Potyvirus* in the family *Potyviridae* [32] with worldwide distribution and infects maize, sorghum, sugarcane, and various grasses in the family *Poaceae* and recently SCMV was reported infecting canna in the family *Cannaceae* [33]. In this study, the genome of SCMV was characterized infecting canna in Beijing and further inoculation experiment showed that SCMV-canna isolate had its own host range and pathogenicity, which was different from the isolate SCMV-BJ. Host range determinants in some potyvirus genomes had been identified. The P3 protein of *Turnip mosaic virus* was an important factor in determining the infection phenotypes in cabbage (*Brassica oleracea* L.) and Japanese radish (*Raphanus sativus* L.) [34]. The amino acid Lys^27^ of *Papaya ringspot virus* NIa-Pro determined the host specificity for its infection of papaya [35]. The P3 and CI cistron played important roles in infectivity of PVY toward *C. annuum* [36]. To determine the key factors of SCMV-canna host specificity, infectious clone of SCMV-canna should be constructed whereas site-directed mutagenesis or chimeric viral infectious clones were used to identify host range determinants.

Sequence comparisons showed SCMV-canna shared the highest 78 and 85% sequence identities at the nt and aa levels with specific SCMV isolates. Furthermore, SCMV-canna shared the highest nt sequence identity in the CP gene (90%) with other known isolates, while 65–80% sequence identities in other genes (The lowest in P1). This was common in plant RNA viruses with high mutation rates due to the base mismatch in replication, short generation time and large population size [37] and genetic recombination which was also detected in this isolate SCMV-canna, one of the important forces shaping the plant RNA virus evolution [38]. Phylogenetic analyses based on the complete genomic nt/aa sequences with different methods revealed that SCMV-canna clustered independently, indicating this isolate a phylogenetically distinct isolate. This was also in accordance with the sequence comparison analyses.

The symptom of mosaic leaves and dwarf plants and severe agronomical losses worldwide were often observed in sugarcane, maize, johnsongrass, millet, sorghum species, and other poaceous plants when infected by SCMV [39]. Inoculation test showed SCMV-canna could infect the selected maize cultivars and *Pennisetum alopecuroides* (L.) Spreng, but the symptoms were less severe than that caused by SCMV-BJ. Furthermore, the low incidence of SCMV-canna in cultivars ludan 6006 and nongda 2238 was also observed (data not shown). Early studies concluded that host range expansion was often traded off against a decreased fitness in the original host species [40–42]. This could explain the fitness of SCMV-canna isolate in different maize plants. Otherwise, the levels of virus-host interactions compatibility affect host physiology and induce disease symptoms with different severity, dependent on the disturbance of plant physiology and development [43]. The SCMV-canna and SCMV-BJ caused different symptoms in different hosts but the underlying mechanisms was unknown, thus the interactive network could be explored together with the approaches for transcriptomic and proteomic analyses available to the research community [44], which will get a global picture of the plant-SCMV interactions that could help clarify the mechanism of SCMV pathogenicity.

In our study, SCMV was identified in nearly all of the canna samples with mosaic symptom. It is common that cannas are propagated via vegetative means and distributed through the frequent exchange of untested material which could disperse and increase the incidence of viral disease. Like all potyviruses, SCMV is transmitted by aphids in a non-persistent manner, thus the potential of the transmission of this isolate to maize or other host plants should be monitored as a mixed infection of different strains could change the virus virulence through recombination [45] which had been detected to play an important role in the evolution of SCMV [46, 47].

## Conclusions

In conclusion, the casual agent of mosaic disease in canna was SCMV, a prevalent virus threatening the canna planting in Beijing. This isolate (SCMV-canna) shared relative low sequence identity (78% nt and 85% aa sequence identities) with SCMV isolates from other hosts and clustered into a separate branch in the phylogenetic tree. Recombinant analysis showed SCMV-canna was a naturally occurred recombinant originated from isolates infecting sugarcane and maize. The pathogenicity of SCMV-canna was relative less than the strain SCMV-BJ. This study enriched the research on viruses infecting canna and provided details on the characterization of SCMV-canna, which was helpful to the management of this disease.

## Methods

### Sample collection, RNA extraction and deep sequencing

In October of 2014, canna plants with mosaic symptom in the leaves were observed in Beijing Botanical Garden. The symptomatic leaves from a plant were randomly collected with the Beijing Botanical Garden′s permission and the formal identification of the plant material was undertaken by the corresponding author of this article. No voucher specimens of this material were deposited. This plant was not protected or endangered species, thus no detailed permission was supplied. The samples were quickly frozen in liquid nitrogen and total RNA was extracted from the samples with TRIzol reagent (Invitrogen, Carlsbad, CA, USA) with standard procedure as recommended by the manufacturer. The quality and quantity of the RNA was assessed using a Nanodrop ND-1000 (Nanodrop Technologies, Wilmington, DE) and Bio-Analyzer 2100 (Agilent Technologies, Waldbronn, Germany). Small RNAs of the length of 18–28 nt were isolated from the total RNA with 15% polyacrylamide gel for 5′ and 3′ adaptors ligation. The final ligation products were purified and reverse-transcribed for sequencing library construction. Deep sequencing was performed according to the manufacturers’ instructions using the Illumina Hiseq2000 sequencing platform.

### Bioinformatic analyses and determination of full-length viral genome

Raw Illumina sRNA reads were trimmed and cleaned by removing the sequences smaller than 16 nucleotides or longer than 30 nucleotides, low-quality tags, polyA or N tags with an in-house perl script. The resulting clean reads were assembled into contigs using the CLC Genomics Workbench which were further analyzed by BLASTn and BLASTx searches against the GenBank database. The overlapped contigs were manually assembled. Primers were then designed to amplify PCR products spanning the gaps between the various contigs and both ends of the virus genome were determined with Rapid Amplification of cDNA Ends (RACE) PCR (Primers were listed in Additional file 3: Table S1).

### Sequence, recombination and phylogenetic analyses

The available SCMV sequences were downloaded from GenBank database and phylogenetic tree was constructed with the aligned sequences with Clustal W [48] using the Neighbor-Joining (NJ) and Maximum-likelihood (ML) methods implemented in the MEGA6.06 program [49] with the best-fit models recommended by a model test in this software. Recombination was detected with various recombination detection methods implemented in the software RDP4 [50] including the programs RDP, GENECONV, BOOTSCAN, MAXCHI, CHIMAERA, SISCAN and 3SEQ, performed with the default configuration with the thirty SCMV complete genome sequences available in GenBank database (Additional file 4: Table S2).

### Prevalence of this virus in canna

To understand the occurrence of viral disease in canna, a survey was conducted in 24 gardens of the Chaoyang (8), Haidian (6), Changping (7) and Yanqing (3) districts of Beijing and 64 (18 from Chaoyang, 16 from Haidian, 22 from Changping and 8 from Yanqing) canna plants with mosaic symptom were collected and preserved in -80 °C. Total RNAs were purified from the leaf tissues with TRIzol reagent (Invitrogen, Carlsbad, CA, USA) according to the manufacturer’s instructions. RT-PCR detection of SCMV was performed by a two-step cDNA reverse transcription with random hexanucleotide and oligo (dT) primers and the following PCR was carried out using SCMV-specific forward and reverse primers designed on sequence of our isolate (Additional file 3: Table S1) with *Taq* DNA polymerase (TaKaRa).

### Infectivity test of SCMV- canna

To test the infectivity of SCMV-canna, the canna leaves with mosaic symptom was homogenized in liquid nitrogen and dissolved in 0.01 mol/l phosphated buffer (pH 7.0) which was then centrifuged for separation of inoculum. Plants in the species of *Nicotiana* (*Nicotiana benthamiana*, *Nicotiana tabacum* (cv. Xanthi nc)), *Chenopodium* (*Chenopodium amaranticolor, C. quinoa*)*, Zea* (cultivars denghai 605, ludan 6006, ludan 9066, luke super maize, jundan 20 and nongda 2238), *Sorghum* (*Sorghum sudanense (Piper) Stapf.*, Sorghum Hybrid sudan grass), *Pennisetum* (*Pennisetum alopecuroides* (L.), *Pennisetum purpureum* Schum.) were selected for pathogenicity test with three independent repeats. Inoculated plants were kept in the greenhouse (28 °C day and 22 °C night, 16 h light and 8 h dark cycles) for symptom observation. All the tested plants were also inoculated with the typical SCMV isolate SCMV-BJ and PBS buffer as control.

## Supplementary information

**Additional file 1:** **Figure S1.** Maximum Likelihood trees obtained from alignment of the complete genome nucleotide sequence (A) and amino acid sequence (B) of SCMV with 1000 bootstrap replicates. Bootstrap values are given by numbers at the relevant nodes in the topology and only bootstrap values of ≥50% were shown. Maize dwarf mosaic virus (MDMV), *Pennisetum mosaic virus* (PenMV), *Canna yellow streak virus* (CaYSV), *Johnsongrass mosaic virus* (JGMV) were used as outgroups.

**Additional file 2:** **Figure S2.** Neighbor-joining tree obtained from alignment of the CP gene of SCMV with 1000 bootstrap replicates. Bootstrap values are given by numbers at the relevant nodes in the topology and only bootstrap values of ≥50% were shown. Maize dwarf mosaic virus (MDMV), *Pennisetum mosaic virus* (PenMV), *Canna yellow streak virus* (CaYSV), *Johnsongrass mosaic virus* (JGMV) were used as outgroups.

**Additional file 3:** **Table S1.** Primers used for RT-PCR and Rapid Amplification of cDNA Ends (RACE) in this study.

**Additional file 4:** **Table S2.** Sources of SCMV isolates used for RDP detection in this study.

## Data Availability

Materials described in the manuscript, including all relevant raw data, will be freely available to any scientist wishing to use them for non-commercial purposes upon request via e-mail with the corresponding author.

## Abbreviations

BYMV: *Bean yellow mosaic virus*
CaYMV: *Canna yellow mottle virus*
CaYSV: *Canna yellow streak virus*
CMV: *Cucumber mosaic virus*
NGS: Next generation sequencing
ORF: Open reading frame
RACE: Rapid Amplification of cDNA Ends
SCMV: *Sugarcane mosaic virus*
TAV: *Tomato aspermy virus*
UTR: Untranslated regions
vsiRNA: Virus-derived small interfering RNA
