# Supplementary material for: Characterization of a highly divergent Sugarcane mosaic virus from Canna indica L. by deep sequencing
Source: BMC Microbiol. 2019 Nov 21;19:260. doi: 10.1186/s12866-019-1636-y (PMC6873528; doi:10.1186/s12866-019-1636-y)
Supplement: Supplementary file 4 — Additional file 4: Table S2. Sources of SCMV isolates used for RDP detection in this study. [file 12866_2019_1636_MOESM4_ESM.doc]

**Table S2.** Sources of SCMV isolates used for RDP detection in this study.

| Series No. | GenBank access. no. | Host | Geographical Origin | Collection time | Reference |
| --- | --- | --- | --- | --- | --- |
| 1 | AF494510.1 | maize | China (Henan) | ----- | Liu *et al.* Acta Virol. 2003, 47:223-227 |
| 2 | EU091075.1 | maize | Mexico (Veracruz) | ----- | Chaves-Bedoya *et al.* Viro. J. 2011, 8:15 |
| 3 | JN021933.1 | maize | China (Hebei) | 2009 | Gao *et al.* Virus Genes 2011, 43:390-397 |
| 4 | AY569692.1 | maize | China (Shanxi) | 1999 | Zhong *et al.* Virus Genes 2005,30:75-83 |
| 5 | AY149118.1 | maize | China (Shandong) | ----- | unpublished |
| 6 | GU474635.1 | maize | Mexico | ----- | unpublished |
| 7 | AJ297628.1 | maize | China (Zhejiang) | 1999-2000 | Chen *et al.* Arch.Virol. 2002,147:1237-1246 |
| 8 | AJ310103.1 | sugarcane | China (Zhejiang) | 1999-2000 | Chen *et al.* Arch.Virol. 2002,147:1237-1246 |
| 9 | AJ310105.1 | maize | China (Guangdong) | 1999-2000 | Chen *et al.* Arch.Virol. 2002,147:1237-1246 |
| 10 | AJ278405.1 | sugarcane | Australia (Brisbane) | ----- | unpublished |
| 11 | AJ310102.1 | sugarcane | China (Zhejiang) | 1999-2000 | Chen *et al.* Arch.Virol. 2002,147:1237-1246 |
| 12 | AJ310104.1 | sugarcane | China (Zhejiang) | 1999-2000 | Chen *et al.* Arch.Virol. 2002,147:1237-1246 |
| 13 | AM110759.1 | maize | Spain | 1998 | Achon *et al* Arch.Virol. 2007, 152:2073-2078 |
| 14 | AY042184.1 | maize | China (Beijing) | ----- | Fan *et al.* Arch. Virol. 2003, 148:773-782 |
| 15 | JX185303.1 | maize | Germany (Seehausen) | ----- | unpublished |
| 16 | KP772216.1 | maize | Ethiopia | 2014 | Mahuku *et al.* Plant Dis. 2015, 99:1870 |
| 17 | KP860936.1 | maize | Ethiopia | 2014 | Mahuku *et al.* Plant Dis. 2015, 99:1870 |
| 18 | KP860935.1 | maize | Ethiopia | 2014 | Mahuku *et al.* Plant Dis. 2015, 99:1870 |
| 19 | KF744390.1 | maize | Rwanda | 2013 | Adams *et al.* New Dis. Rep. 2014, 29: 22 |
| 20 | KF744391.1 | maize | Rwanda | 2013 | Adams *et al.* New Dis. Rep. 2014, 29: 22 |
| 21 | KF744392.1 | maize | Rwanda | 2013 | Adams *et al.* New Dis. Rep. 2014, 29: 22 |
| 22 | JX188385.1 | maize | USA | 1965 | unpublished |
| 23 | KR108212.1 | sugarcane | China | 2014 | unpublished |
| 24 | KR108213.1 | sugarcane | China | 2014 | unpublished |
| 25 | JX237862.1 | sugarcane | Argentina | 2010 | unpublished |
| 26 | JX237863.1 | sugarcane | Argentina | 2007 | unpublished |
| 27 | KT895080.1 | sugarcane | Iran (Mazandaran) | 2013 | Moradi *et al.* Virus Genes 2016, 52:270-280 |
| 28 | KT895081.1 | maize | Iran (Mazandaran) | 2013 | Moradi *et al.* Virus Genes 2016, 52:270-280 |
| 29 | KY006657.1 | maize | Ecuador | 2016 | Moradi *et al.* Virus Genes 2016, 52:270-280 |
| 30 | KU561096.1 | canna | China (Beijing) | 2014 | This study |

Note: “-----“ indicates that the relative information was not available.
