# Supplementary material for: Characterization of a highly divergent Sugarcane mosaic virus from Canna indica L. by deep sequencing
Source: BMC Microbiol. 2019 Nov 21;19:260. doi: 10.1186/s12866-019-1636-y (PMC6873528; doi:10.1186/s12866-019-1636-y)
Supplement: Supplementary file 3 [file 12866_2019_1636_MOESM2_ESM.docx]

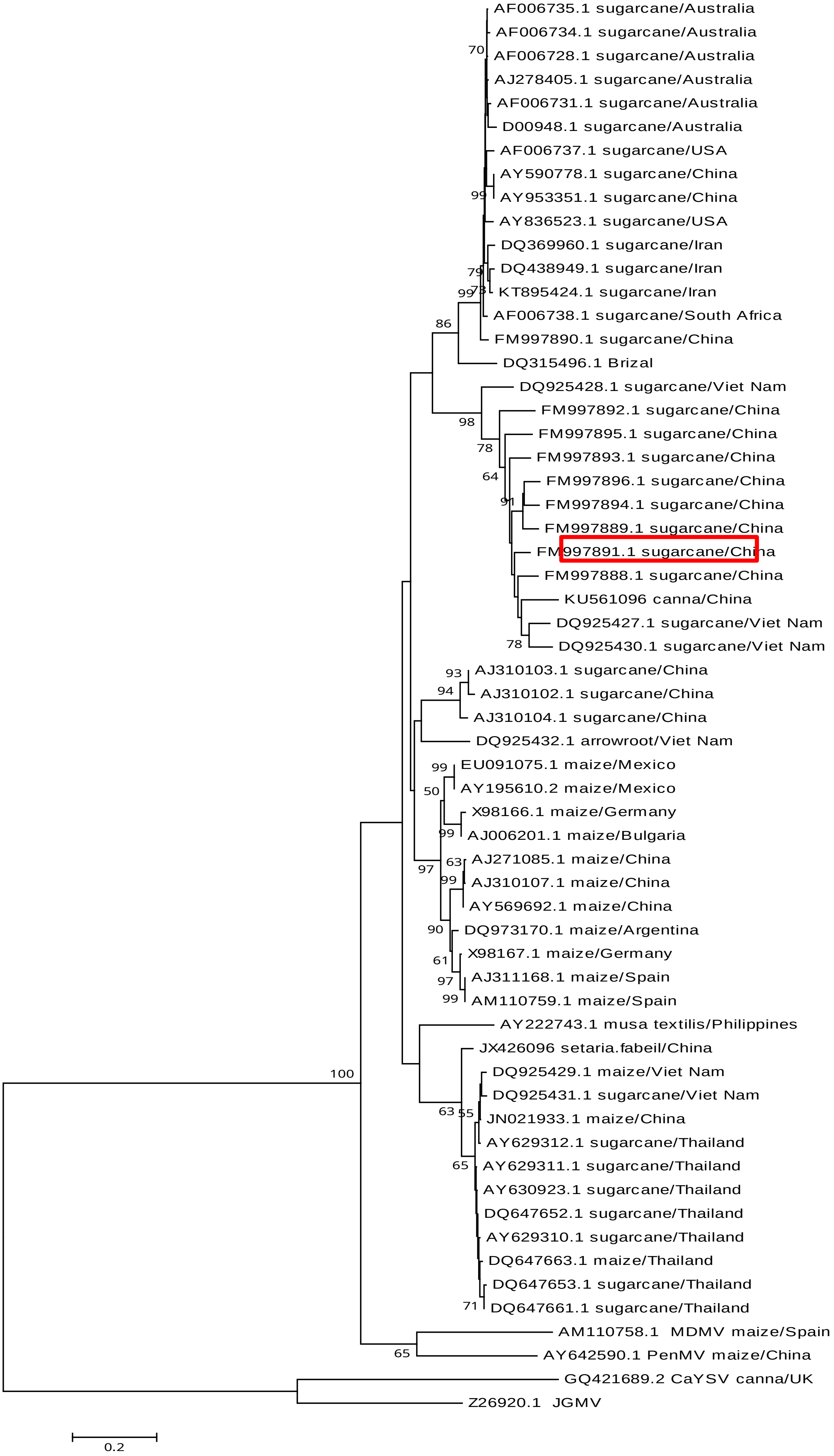
AF006735.1 sugarcane/Australia
AF006734.1 sugarcane/Australia
AF006728.1 sugarcane/Australia
AJ278405.1 sugarcane/Australia
AF006731.1 sugarcane/Australia
D00948.1 sugarcane/Australia
AF006737.1 sugarcane/USA
AY590778.1 sugarcane/China
AY953351.1 sugarcane/China
AY836523.1 sugarcane/USA
DQ369960.1 sugarcane/Iran
DQ438949.1 sugarcane/Iran
KT895424.1 sugarcane/Iran
AF006738.1 sugarcane/South Africa
FM997890.1 sugarcane/China
DQ315496.1 Brizal
DQ925428.1 sugarcane/Viet Nam
FM997892.1 sugarcane/China
FM997895.1 sugarcane/China
FM997893.1 sugarcane/China
FM997896.1 sugarcane/China
FM997894.1 sugarcane/China
FM997889.1 sugarcane/China
FM997891.1 sugarcane/China
FM997888.1 sugarcane/China
KU561096 canna/China
DQ925427.1 sugarcane/Viet Nam
DQ925430.1 sugarcane/Viet Nam
AJ310103.1 sugarcane/China
AJ310102.1 sugarcane/China
AJ310104.1 sugarcane/China
DQ925432.1 arrowroot/Viet Nam
EU091075.1 maize/Mexico
AY195610.2 maize/Mexico
X98166.1 maize/Germany
AJ006201.1 maize/Bulgaria
AJ271085.1 maize/China
AJ310107.1 maize/China
AY569692.1 maize/China
DQ973170.1 maize/Argentina
X98167.1 maize/Germany
AJ311168.1 maize/Spain
AM110759.1 maize/Spain
AY222743.1 musa textilis/Philippines
JX426096 setaria.fabeil/China
DQ925429.1 maize/Viet Nam
DQ925431.1 sugarcane/Viet Nam
JN021933.1 maize/China
AY629312.1 sugarcane/Thailand
AY629311.1 sugarcane/Thailand
AY630923.1 sugarcane/Thailand
DQ647652.1 sugarcane/Thailand
AY629310.1 sugarcane/Thailand
DQ647663.1 maize/Thailand
DQ647653.1 sugarcane/Thailand
DQ647661.1 sugarcane/Thailand
AM110758.1 MDMV maize/Spain
AY642590.1 PenMV maize/China
GQ421689.2 CaYSV canna/UK
Z26920.1 JGMV
0.2
